# Upregulation of miR-361-5p attenuates MPP^+^-induced neurotoxicity in dopaminergic SH-SY5Y cells as Parkinson's disease model

**DOI:** 10.1016/j.jbc.2025.110912

**Published:** 2025-11-05

**Authors:** Shiva Vaheb Hosseinabadi, Hananeh Tahamtan, Maryam Mahmoudian Esfahani, Rasoul Ghaedi-Heidari, Motahare-Sadat Hashemi, Maryam Peymani, Sajjad Sisakhtnezhad, Hossein Fahimi, Kamran Ghaedi

**Affiliations:** 1 Department of Biology, Faculty of Science, Razi University, Kermanshah, Iran; 2 Department of Genetics, Faculty of Advanced Science and Technology, Tehran Medical Sciences, Islamic Azad University, Tehran, Iran; 3 Department of Biology, Faculty of Science, NourDanesh Institute of Higher Education, Isfahan, Iran; 4 Department of Biology, Faculty of Science, Hakim Sabzevari University, Sabzevar, Iran; 5 Department of Animal Biotechnology, Cell Science Research Center, Royan Institute for Biotechnology, ACECR, Isfahan, Iran; 6 Department of Biology, ShK.C., Islamic Azad University, Shahrekord, Iran; 7 Department of Cell and Molecular Biology and Microbiology, Faculty of Biological Science and Technology, University of Isfahan, Isfahan, Iran

**Keywords:** Parkinson's disease, mir-361-5p, *HMOX1,* FADD, apoptosis, ferroptosis

## Abstract

Programmed cell death plays a crucial role in the pathogenesis of Parkinson's disease, yet the involvement of microRNAs (miRNAs) in this process remains inadequately understood. Previous studies have indicated a decreased expression pattern of hsa-miR-361-5p in patients with neurodegenerative diseases and in cell models of these conditions. This study aimed to predict the target genes of hsa-miR-361-5p, and to validate the predicted interactions at the mRNA level between hsa-miR-361-5p and its predicted target genes. In addition, we investigated the potential neuroprotective effect of miR-361-5p by assessing its effects on apoptosis and cell viability in SHSY-5Y cells using MTS assay and flow cytometry. Notably, overexpression of miR-361-5p significantly attenuated MPP^+^-induced neurotoxicity. Utilizing bioinformatics and dual-luciferase reporter assays, heme oxygenase 1 (HMOX1) and Fas associated via death domain (FADD) were identified as direct targets of miR-361-5p. Moreover, overexpression of miR-361-5p led to a decrease in the expression of *HMOX1* and *FADD* genes, both known to be involved in cell death processes such as apoptosis and ferroptosis. Our findings indicate that miR-361-5p regulates the expression of *HMOX1* and *FADD*, suggesting that miR-361-5p may represent a potential therapeutic target in Parkinson's disease.

Mitochondrial failure and oxidative stress are significant players responsible for neuronal cell demise and are strongly connected to neurodegenerative diseases (1). Oxidative injury to mitochondrial function yields increased reactive oxygen species (ROS) production, energy loss, and the generation of apoptotic mechanisms in neuronal cells. These cellular events are most applicable to Parkinson's disease (PD), a progressive neurodegenerative illness distinguished by the degeneration of substantia nigra-based dopaminergic neurons (2). PD is the second most common neurodegenerative condition worldwide, following Alzheimer's disease (3). It is primarily associated with aging; while it is rare to find cases occurring in individual younger than 40 years, around 2 percent of those over 60 years are diagnosed with it (4, 5). At its core, PD is a chronic disorder that affects specific brain cells called dopaminergic neurons, particularly in an area known as the pars compacta of the corpus striatum (6, 7). As the disease progresses, the levels of dopamine, a crucial neurotransmitter, start to decline, leading to symptoms like motor symptoms such as tremors, rigidity, and bradykinesia (8, 9). One of the major factors contributing to PD is mitochondrial dysfunction, which can trigger oxidative stress and ultimately cause the death of these important neurons (10). Therefore, the key factors that disrupt mitochondrial function in cellular PD models may provide avenues to develop diagnostic markers and therapeutic strategies.

MicroRNAs (miRNAs) are defined as a group of endogenous small noncoding RNAs that regulate the expression of their target genes at the posttranscriptional level. The binding of miRNAs to the seed region, typically located in the 3′untranslated region (3′UTR) of target messenger RNA (mRNA), mediates mRNA degradation or translational repression (11, 12). Research has shown that miRNAs are vital for the survival and proper functioning of neurons. When their expression levels are dysregulated, it can contribute to various neurodegenerative diseases, including multiple sclerosis, Alzheimer's, and PD (13). Therefore, miRNAs have been suggested as novel diagnostic markers or even therapeutic agents for future clinical applications. One particular miRNA, miR-361-5p, has been implicated in processes such as apoptosis and inflammation, both of which are tightly associated with PD. Furthermore, several studies have shown that decreased miR-361-5p expression is correlated with neurodegenerative and age-related diseases, comprising Alzheimer's, major depressive disorder, and age-related macular degeneration (AMD) (14, 15).

The heme oxygenase 1 *(HMOX1)* gene encodes a key enzyme in heme catabolism, which catalyzes the degradation of free heme into biliverdin, carbon monoxide, and ferrous iron (16). *HMOX1* expression can be upregulated in response to aging and various cellular stressors, which in turn may exacerbate oxidative stress, a significant contributor to PD cell model pathogenesis (17). Research has shown an abnormal accumulation of iron levels within dopaminergic neurons and glial cells in patients with PD. Moreover, recent findings indicate that excess iron in cells can trigger a specific type of programmed cell death known as ferroptosis. This process has been implicated in the pathology of various neurological disorders, including PD (18, 19).

The Fas associated via death domain *(FADD*) gene encodes an adaptor protein that interacts with various cell surface receptors to mediate apoptotic signaling pathways (20). Interaction between *FADD and* leucine rich repeat kinase 2 (LRRK2), a known contributor to PD cell model pathology, is required for the activation of caspase-8 and the subsequent induction of programmed cell death (21). Although substantial progress has been made in defining the increased expression of *HMOX1* and *FADD* in PD patients, the possible underlying mechanisms of their expression alterations during PD pathogenesis remain largely unknown and warrant further investigation.

1-Methyl-4-phenylpyridinium (MPP^+^) is a widely used neurotoxin to establish mitochondrial dysfunction models (22). Upon entering mitochondria, MPP^+^ binds to the same site as rotenone, a well-known inhibitor of mitochondrial complex I (23). The resulting decrease in ATP levels leads to the sudden release of neurotransmitters by causing a failure in the electrogenic pumps (24). MPP^+^ has also been reported to inhibit mitochondrial complexes III and IV (25), and subsequent increased levels of ROS induce mitochondrial dysfunction through the transient permeability of mitochondrial membranes, release of cytochrome C from the mitochondria into the cytoplasm, and activation of caspases-3 and caspases-9 (26). Furthermore, as deregulated expression of miRNAs can profoundly affect the expression of PD-related genes (13), we planned to explore the direct interaction of miR-361-5p with the *HMOX1* and *FADD* genes.

In this investigation, we initially detected an inverse correlation between the expression levels of *FADD* and *HMOX1* and miR-361-5p in SH-SY5Y cells relative to control conditions. Subsequent validation identified *HMOX1* and *FADD* as direct targets of miR-361-5p. Furthermore, overexpression of miR-361-5p reduced the mRNA levels of *FADD* and *HMOX1*. Notably, our findings demonstrated that increased miR-361-5p expression enhanced cell viability while mitigating ROS production and apoptosis. These cellular results suggest a potential regulatory mechanism involving miR-361-5p axis in cellular processes, suggesting a mechanism relevant to PD cell model pathogenesis and a potential avenue for therapeutic development.

## Results

### Downregulation of miR-361-5p expression in response to MPP^+^ treatment

**Figure 1:**
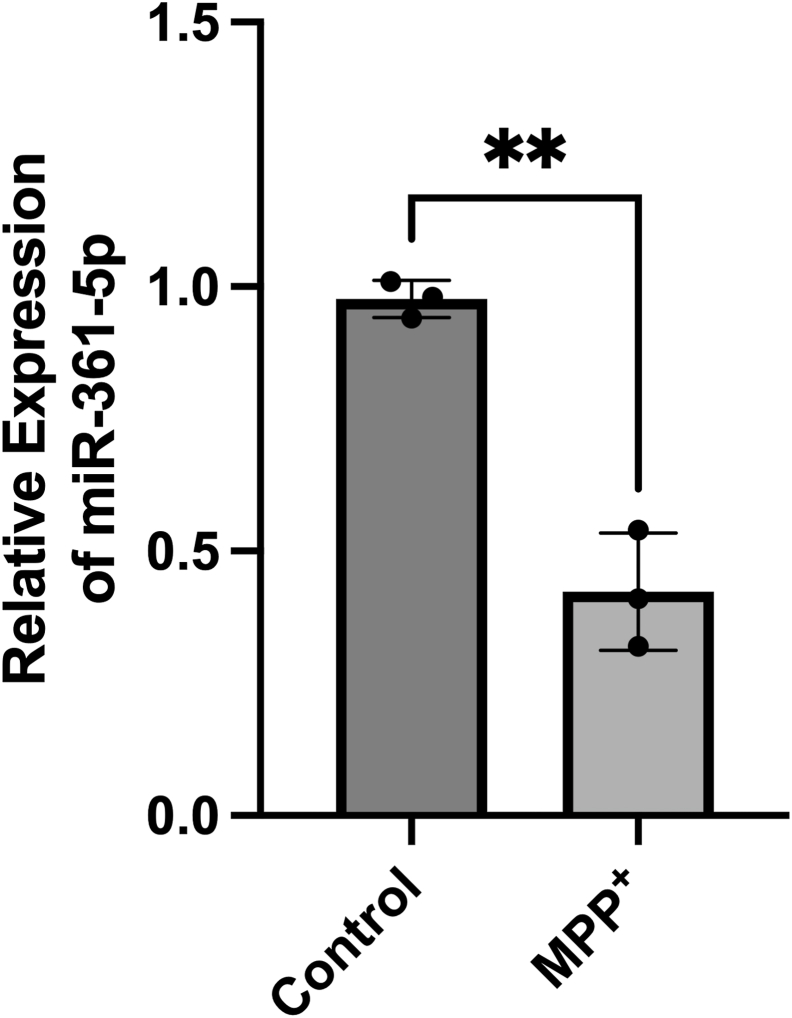
**Deregulation of mir-361-5p in MPP^+^-treated SHSY-5Y cells**. RT-qPCR analysis of miR-361-5p in MPP + -treated and untreated SHSY-5Y cells. Results are expressed as mean ± SEM from at least three independent experiments performed in technical triplicates. Statistical comparisons were carried out using Student’s *t* test (∗∗*p* < 0.01). Scatter plots indicate individual data points. MPP^+^, 1-Methyl-4-phenylpyridinium; RT-qPCR, quantitative real-time PCR.

To investigate whether mitochondrial stress affects miR-361-5p expression, SH-SY5Y cells were treated with MPP+ (2 mM). The expression changes were normalized to U6 snRNA, serving as a reliable internal control for assessing miRNA expression in SH-SY5Y cells. Quantitative analysis of miRNA expression through quantitative real-time PCR (RT-qPCR) revealed a significant reduction in miR-361-5p levels in cells treated with MPP^+^ (Fig. 1). This reduction suggests that mitochondrial stress may downregulate miR-361-5p, potentially affecting the regulation of its target genes under neurotoxic conditions.

### MiR-361-5p target analysis

**Figure 2:**
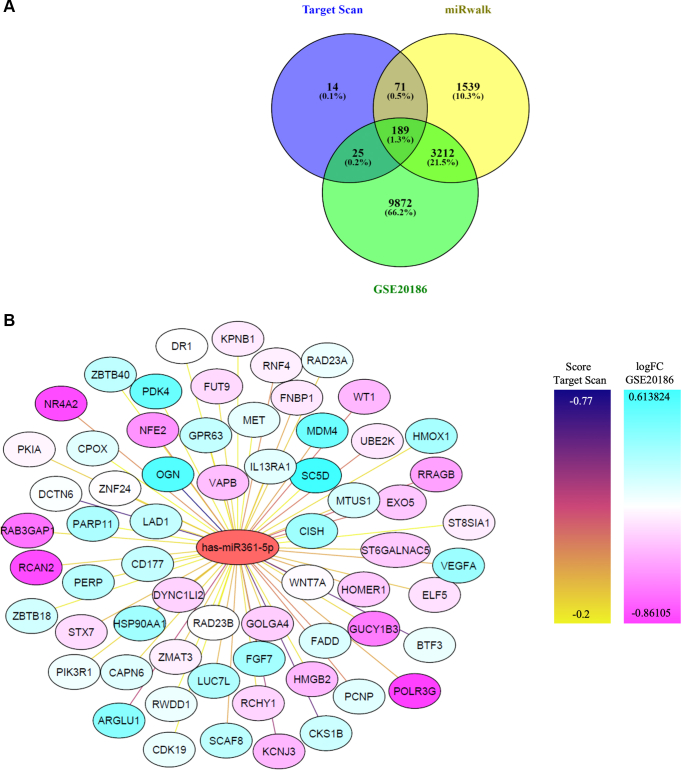
**Genes selection**. *A*,venn diagrams were recruited to obtain the common genes which either involved in Parkinson disease in GEO database or genes target to mir-361-5p in targetscan and mirwalk database. Numbers demonstrate the quantity of genes which in each database. One hundred eighty-nine (1.3%) genes were common between three databases and selected for more analyses. *B*, cytoscape diagram was used for selecting target genes of mir-361-5p. In this diagram, line's color indicates targetscan score for each gene that as the color goes from *yellow* to *purple*, they have a more negative score and another filter for these is log foldChange. In this filter, *blue* color means increased expression of that gene in Parkinson's disease.

To identify potential targets of miR-361-5p that may mediate its role in neuronal stress, we utilized two target gene prediction algorithms, TargetScan and miRWalk, to identify potential target genes of miR-361-5p. Subsequently, we examined the expression profiles of genes associated with PD using a microarray study from the GEO database (GSE20186), comparing individuals with PD to healthy controls. Through this analysis, we identified 189 common genes among the predicted targets of miR-361-5p and genes altered in PD (Fig. 2*A*), of which 65 were retained for further consideration. Further analysis was conducted using the Cytoscape program, focusing on gene expression profiles and the Total Context++ scores from TargetScan, as well as examining signaling pathways using STRING and Kyoto Encyclopedia of Genes and Genomes (KEGG) databases. Ultimately, HMOX1 and FADD were selected for further investigation based on their highest target prediction scores and their convergence within cell death pathways, indicating strong functional relevance. Furthermore, the lack of prior experimental validation for these miRNA–gene interactions justified their selection as suitable candidates for our study, ensuring a data-driven approach that addresses critical knowledge gaps (Fig. 2*B*). These analyses prioritized HMOX1 and FADD as functionally relevant and experimentally unvalidated targets for further investigation.

### Increased expression of *FADD* and *HMOX1* following MPP ^+^ exposure

**Figure 3:**
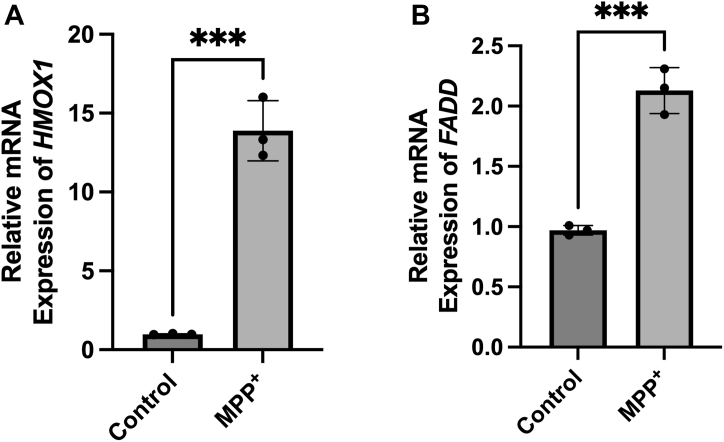
**Deregulation of *HMOX1*, and *FADD* in MPP ^+^ -treated SHSY-5Y cells**. *A*, RT-qPCR analysis of *HMOX1* transcripts expression level in MPP ^+^ -treated and untreated SHSY-5Y cells. *B*, RT-qPCR analysis of *FADD* transcripts expression level in MPP ^+^ -treated and untreated SHSY-5Y cells. Transcript levels were normalized to the expression level of *GAPDH* as the reference gene, and miRNA level was normalized to expression level of the U6 snRNA as the reference gene. Mean ± SEM values from ≥3 biological replicates are presented. Student’s *t* test was used for two-group comparisons. Individual data points are displayed on the bar graphs (∗∗∗*p* < 0.001, versus control). HMOX1, heme oxygenase 1; FADD, Fas associated via death domain; MPP^+^, 1-Methyl-4-phenylpyridinium; RT-qPCR, quantitative real-time PCR.

To determine whether MPP + induces the expression of candidate target genes, RT-qPCR analysis was conducted, with the expression levels normalized to GAPDH as a reference gene. The transcript level of *HMOX1* exhibited a notable increase after 24 h of treatment with MPP^+^ (2 mM) in SH-SY5Y cells, as compared to the control group. Similarly, the transcript levels of *FADD* also showed a significant rise following exposure to MPP^+^ (Fig. 3, *A* and *B*). These findings indicate that MPP + -induced mitochondrial stress upregulates HMOX1 and FADD, which may contribute to cellular stress responses. These transcriptional changes provide important insights into the regulation of HMOX1 and FADD under mitochondrial stress, forming a basis for future protein-level validation.

### The miR-361-5p directly targets the 3′UTR of *HMOX1* and FADD

**Figure 4:**
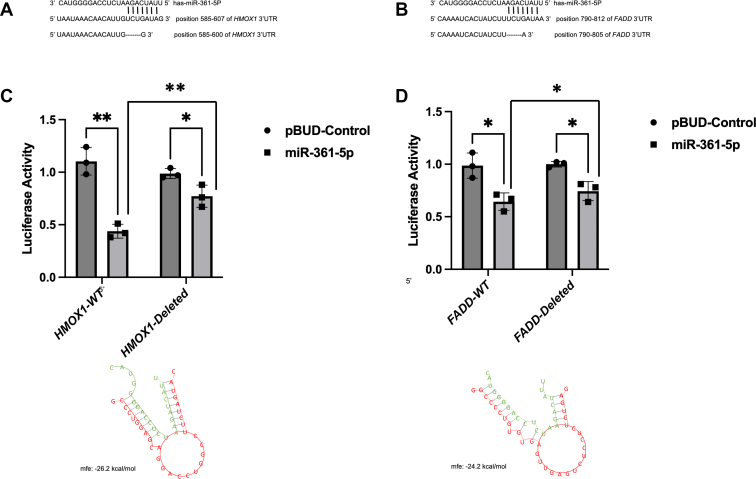
**miR-361-5p directly targets the 3′untranslated region (UTR) of *HMOX1* and FADD**. *A*, miR-361-5p-binding site predictions for *HMOX1. B*, miR-361-5p-binding site predictions for *FADD*. target validation using dual-luciferase reporter method in HEK293T cells. *C*, relative luciferase activity of *HMOX1* luciferase vectors containing wild-type (WT) or mutant (MT) transcripts was measured after cotransfection with the indicated miRNA. *D*, relative luciferase activity of *FADD*. Results are presented as mean ± SEM, based on three independent experiments conducted in triplicate. Comparisons between two groups were analyzed using Student’s *t* test (∗*p* < 0.05, ∗∗*p* < 0.01). Replicate data are overlaid on the bars. HMOX1, heme oxygenase 1; FADD, Fas associated via death domain.

To investigate the direct interaction between miR-361-5p and *FADD* and *HMOX1*, we employed a reporter assay. In this assay, luciferase activity was measured following the cotransfection of either miR-361-5p mimic or NC-mimic with the psichek2 vector containing either the WT or deleted seed region of *FADD* and *HMOX1* 3′UTR in the SH-SY5Y cell line. The binding site was predicted using TargetScan 7.2 (Fig. 4, *A* and *B*). Remarkably, overexpression of miR-361-5p led to a significant reduction in luciferase activity, approximately two-fold lower compared to controls (Fig. 4, *C* and *D*). This observation supports the direct interaction between miR-361-5p and the *FADD* and *HMOX1* transcripts. This result confirms that miR-361-5p can directly bind to and regulate HMOX1 and FADD transcripts.

### Cell viability after MPP^+^ treatment

**Figure 5:**
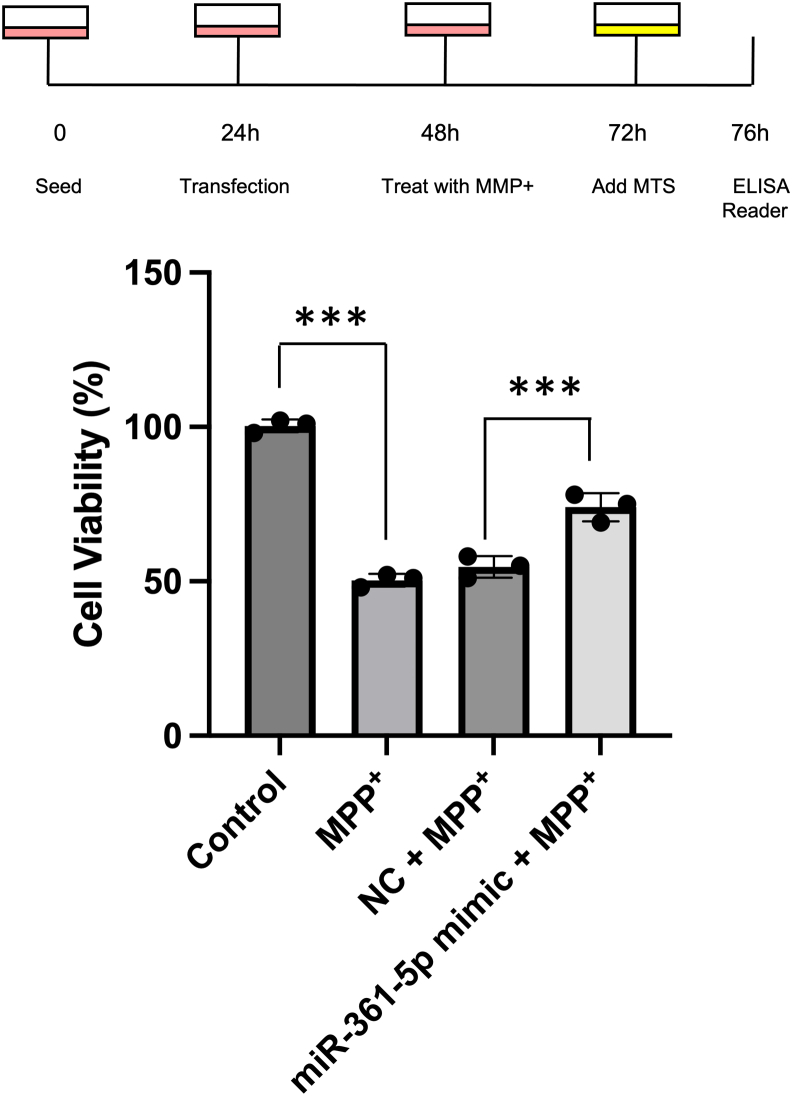
**Cell viability**. SHSY-5Y cells were exposed in negative control, mir-361-5p mimic samples to concentration 2 mM of MPP^+^ for 24 h. Cell viability was monitored using MTS assay. The cell viability rate in mir-361-5p mimic sample has increased significantly compared to the negative control and the cell sample of Parkinson's disease (MPP^+^). All data are expressed as mean ± SEM from at least three independent biological experiments. Statistical analyses were performed by one-way ANOVA followed by Tukey’s multiple-comparison test. Each dot represents one biological replicate (∗∗∗*p* < 0.001). MTS, 3-(4,5-dimethylthiazol-2-yl)-5-(3-carboxymethoxyphenyl)-2-(4-sulfophenyl)-2H-tetrazolium; MPP+, 1-Methyl-4-phenylpyridinium.

To evaluate the functional impact of miR-361-5p on cell survival under mitochondrial stress, SH-SY5Y cells were exposed to MPP+ (2 mM) and cell viability was assessed using the [3-(4,5-dimethyl-thiazol-2-yl)-5-(3-carboxymethoxy phenyl)-2-(4-sulfophenyl)-2H-tetrazolium (MTS) assay. MPP + treatment caused a significant reduction in cell viability compared to the control group. Cells transfected with miR-361-5p mimic showed increased viability 24 h post treatment, reaching approximately 76%, compared to those treated with MPP + alone or with negative control (NC) mimic, indicating a protective effect of miR-361-5p against MPP + -induced neurotoxicity. These findings indicate that upregulation of miR-361-5p exerts a protective effect against MPP^+^-induced neurotoxicity (Fig. 5).

### MiR-361-5p regulates the expression of *HMOX1* and *FADD* in SH-SY5Y cells

**Figure 6:**
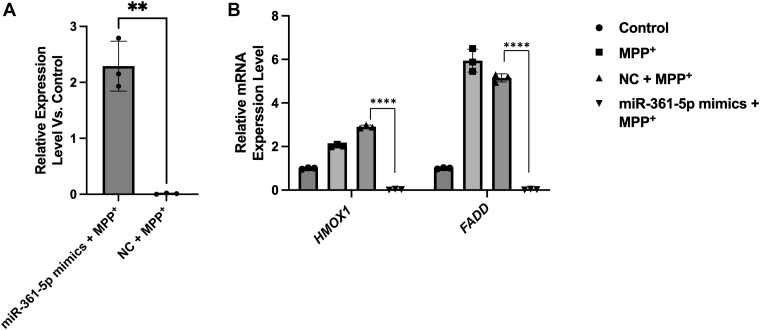
**miR-361-5p regulates the expression of *HM*OX1 and FADD**. *A*, RT-qPCR analysis of mir-361-5p in MPP + -treated cells transfected with mimic of this miRNA and negative control. *B*, *HMOX1* and FADD mRNA expression in cells transfected with miR-361-5p negative control or mimics. Cells were pretreated with miRNAs for 24 h followed by exposure to MPP for a further 24 h. Data are mean ± SEM (n ≥ 3). Statistics: *t* test (2 groups) or one-way ANOVA with Tukey’s test (≥3 groups). Individual points shown (∗∗*p* < 0.01 and ∗∗∗∗*p* < 0.0001, versus NC + MPP^+^). HMOX1, heme oxygenase 1; FADD*,* Fas associated via death domain; MPP^+^, 1-Methyl-4-phenylpyridinium; NC, negative control; RT-qPCR**,** quantitative real-time PCR.

To investigate the regulatory effect of miR-361-5p on its target genes, HMOX1 and FADD, we examined their mRNA levels after transfection with miR-361-5p mimics in MPP + -treated SH-SY5Y cells. Given the established targeting relationship between miR-361-5p and the 3′UTR of *HMOX1* and *FADD* genes, it is reasonable to infer that miR-361-5p plays a regulatory role in the expression of both genes. To investigate this regulatory function, the expression of *HMOX1* and *FADD* genes was examined after treatment with miR-361-5p mimic and NC mimic in MPP^+^-treated SH-SY5Y cells (2 mM). In the SH-SY5Y cell line exposed to MPP^+^, there was a significant increase in the transcription level of miR-361-5p upon introduction of the miR-361–5p mimic, contrasting with the NC-mimic group (Fig. 6*A*). The quantitative PCR results further indicated that the mRNA expression levels of *HMOX1* and *FADD* were reduced in the presence of miR-361-5p mimics compared to samples treated with NC and MPP^+^ (Fig. 6*B*). These findings support the regulatory role of miR-361-5p in suppressing HMOX1 and FADD expression under mitochondrial stress. Although protein-level validation is pending, these results support the regulatory role of miR-361-5p at the transcriptional level under mitochondrial stress conditions.

### The upregulation of miR-361-5p has been shown to effectively inhibit MPP + -induced neurotoxicity

**Figure 7:**
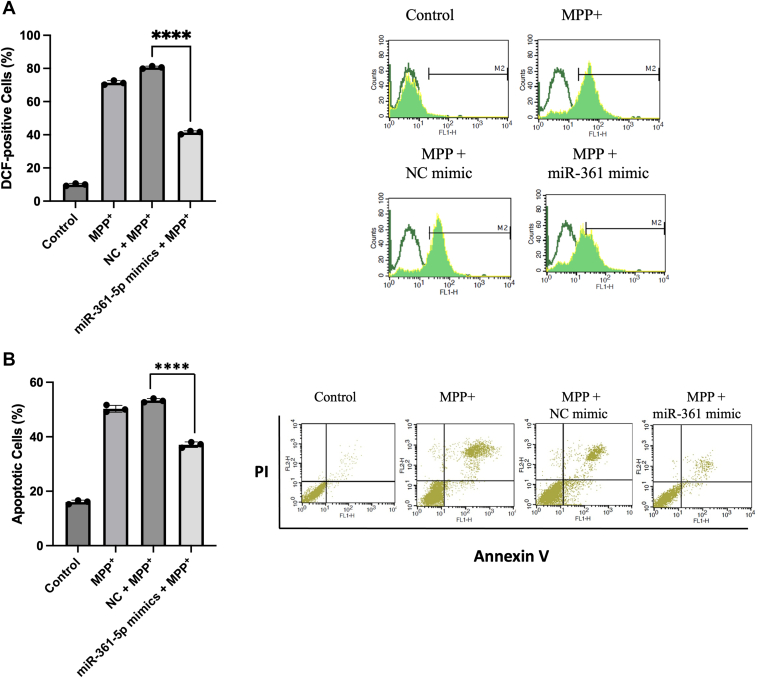
**Detection of the effect of miR-361-5p on MPP^+^-induced neurotoxicity**. *A*, flow cytometric analysis of ROS with DCFH-DA staining. MPP+ (2 mM, 24 h) increased ROS-positive cells from ∼10% to ∼80% (∼8-fold), while miR-361-5p mimic reduced this to ∼45% (∼44% decrease versus MPP^+^). *B*, apoptosis measured by Annexin V-FITC/PI staining. MPP^+^ markedly increased apoptotic cells, which were significantly reduced by miR-361-5p mimic. Data are mean ± SEM from ≥3 independent experiments with technical triplicates. One-way ANOVA with Tukey’s test was used (∗∗∗∗*p* < 0.0001). Individual data points are shown on bar graphs. ROS, reactive oxygen species; MPP^+^, 1-Methyl-4-phenylpyridinium; DCFH-DA, 2′,7′-dichlorofluorescein diacetate.

To further clarify the protective role of miR-361-5p, we assessed its impact on ROS production and apoptotic cell death in SH-SY5Y cells exposed to MPP. The upregulation of miR-361-5p was shown to effectively inhibit MPP^+^-induced neurotoxicity. In order to reveal the definite role of miR-361-5p, we investigated its effect on ROS production and apoptotic cell death in SH-SY5Y cells treated with MPP^+^ (2 mM). Flow cytometric analysis using 2′,7′-dichlorofluorescein diacetate (DCFH-DA) staining showed that MPP^+^ treatment significantly increased intracellular ROS levels compared to the control group (∼8 fold), which was effectively reduced in cells transfected with the miR-361-5p mimic compared to the MPP^+^ group approximately 44% (Fig. 7*A*).

To further elucidate the role of miR-361-5p in MPP^+^-induced neurotoxicity, we investigated its effect on apoptotic cell death through flow cytometry using Annexin V-FITC staining (Fig. 7*B*). The rate of apoptotic cell death in MPP^+^-treated cells significantly increased compared to the control group, reaching approximately 54%, a phenomenon effectively attenuated in the miR-361-5p mimic group to approximately 39%. Overall, these results indicate that miR-361-5p attenuates ROS accumulation and apoptosis, further confirming its protective role against MPP + -induced neurotoxicity.

## Discussion

In this study, we demonstrated that in an acute PD cell model, exposure of SH-SY5Y cells to 2 mM MPP^+^ for 24 h significantly increased the expression levels of the *HMOX1* and *FADD* genes. In addition, we demonstrated that miR-361-5p directly binds to the 3′UTRs of *HMOX1* and *FADD* using dual-luciferase reporter assays, confirming that miR-361-5p regulates the expression of these genes. Consequently, decreased levels of miR-361-5p led to increased expression of *HMOX1* and FADD, resulting in promoting ROS production and apoptosis.

Given the challenges in accessing human brain tissue from patients, researchers often rely on cell and animal models to unravel the underlying mechanisms of PD cell model. In our investigation, we employed an *in vitro* PD model induced by chronic neurotoxin exposure, which recapitulates key pathological features, including oxidative stress (27). Mitochondrial dysfunction is a hallmark of PD (28), and the neurotoxin 1- methyl-4-phenyl-1,2,3,6-tetrahydropyridine (MPTP), along with its active metabolite MPP+, induces apoptotic cell death by inhibiting complex I, subsequently leading to mitochondrial dysfunction and oxidative stress (29). Consequently, MPP^+^ has been extensively utilized to establish various PD models and to investigate the molecular mechanisms underlying neuronal injury and degeneration. In our research, we initially noted a decline in cellular viability in response to MPP^+^ treatment, correlating with dosage. After careful consideration, we opted for a concentration of 2 mM, resulting in a notable 48.6% decrease in cell survival. This concentration has been previously reported for establishing PD models in SH-SY5Y cells (30).

hsa-miR-361-5p has been associated with various neurodegenerative diseases, including Alzheimer's disease, neuropsychiatric conditions such as major depressive disorder, and age-related conditions, including AMD (macular degeneration) (31, 32). It participates in pathways associated with apoptosis and inflammation, both of which are significant pathways involved in PD cell model pathology (15). Studies investigating miR-361-5p expression in age-related diseases like AMD have consistently reported a significant downregulation. Similarly, research on neurodegenerative diseases has also revealed a decrease in miR-361-5p expression (31, 32). Reduced levels of this miRNA have also been observed in the gray matter of Alzheimer's patients' brains (12). Investigations into the role of miR-361-5p in Parkinson's disease-related genes, such as ROCK1, have demonstrated a decrease in its expression (33, 34). Moreover, miR-361 expression has been notably diminished in dopamine neurons in both young and old mice (35). In addition, a study analyzing serum miR-361 levels in patients revealed a significant downregulation in the early stages relative to controls, whereas its levels were elevated in advanced stages compared to the initial phase (36). In our current study, we observed a substantial reduction in miR-361-5p levels in dopaminergic SH-SY5Y cells treated with MPP^+^ (2 mM).

The pathogenic role of HO-1 is supported by multiple lines of evidence. Firstly, the deletion of *HMOX1* in mice exacerbates the severity of various experimental diseases. Secondly, the incidence or severity of many human diseases correlates with a microsatellite polymorphism in the *HMOX1* promoter, which governs HO-1 expression. Thirdly, drug-induced HO-1 or administration of iron catabolism products has been shown to exert therapeutic effects across multiple diseases (37). Factors such as aging and stress lead to upregulation of HO-1 enzyme levels, leading to elevated free iron and carbon monoxide, thereby amplifying oxidative stress. This process impacts mitochondrial activity and factors like alpha-synuclein accumulation, which are implicated in PD pathogenesis. Hence, HO-1 elevation plays a central role in mediating oxidative stress in PD (38).

Furthermore, the *HMOX1* gene has been identified to play a significant role in the ferroptosis pathway (39). Conversely, the involvement of miR-361-5P in the apoptotic pathway suggests a potential association between miR-361-5P and the selected gene (14, 40). Based on the findings from *in vivo* and *in vitro* studies, both *HMOX1* and *FADD* genes are considered promising therapeutic targets for neurodegenerative diseases. Thus, comprehending the molecular mechanisms through which *HMOX1* and *FADD* exert their neuroprotective effects is imperative for developing effective therapies against these conditions. Recent studies have highlighted the critical role of *HMOX1* as a regulator of the ferroptosis pathway. Under stress conditions, the *HMOX1* gene is upregulated, leading to increased Fe2+ production. Subsequently, Fe2+ amplifies ROS levels *via* the Fenton reaction and enters the mitochondria through FtMt, a mitochondrial iron transporter. This process triggers macroautophagy, which in turn diminishes ATP production, consequently inducing ROS-mediated cell demise, a characteristic feature observed in neurodegenerative diseases, including Parkinson's (38, 41).

The *FADD* gene is activated by receptors, including TNFRSF6/FAS, thereby initiating programmed cell death *via* the extrinsic pathway. Ultimately, the *FADD* protein activates caspase-8, which in turn triggers the caspase cascade and eventually leads to cell death. In addition, the interaction of this gene with LRRK2, a crucial gene in PD and the most common genetic cause thereof, is noteworthy. LRRK2 enhances caspase-8 activity through *FADD* gene. Previous studies have indicated that *FADD* gene expression is elevated in Parkinson's and Alzheimer's diseases, and other neurodegenerative disorders (42).

**Figure 8:**
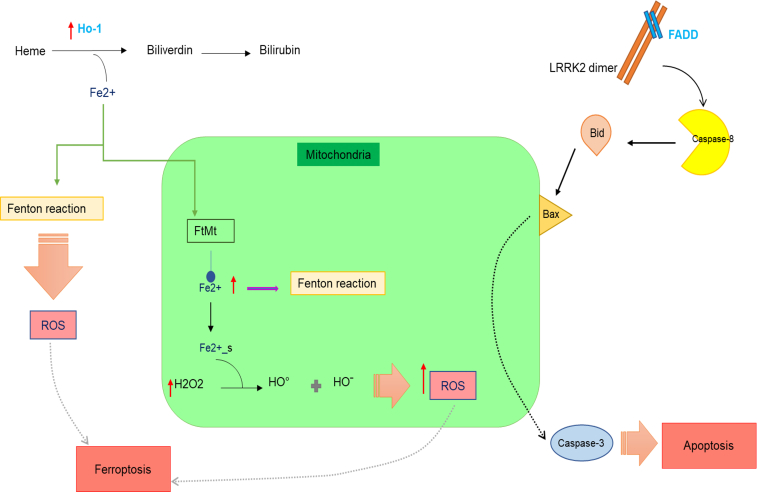
**A schematic overview of apoptosis and ferroptosis pathways at the gene level**. The roles of *HMOX1* and *FADD* within apoptosis/ferroptosis pathways. HMOX1, heme oxygenase ; FADD, Fas associated via death domain*.*

LRRK2 interacts with complex II components, including TNFR1-associated death domain protein (TRADD), TRAF6, and RIP in a kinase-dependent manner *via* FADD. Upon cleavage, caspase-8 is activated, which in turn cleaves and activates caspase-3. In some cells, this is adequate to induce cell death, while others require signal amplification from Bid cleavage, a proapoptotic protein of the Bcl-2 family. Activated Bid translocates to the mitochondria, where it promotes the formation of BAX-BAK oligomers, forming a proteolipid pore. This process releases cytochrome c and other factors from the intermembrane space into the cytosol, activating apoptosis. Caspase-9 activation promotes structural alterations and initiates the proteolytic cascade. Caspase-3 deficiency impairs apoptosis, which may compromise normal cell death pathways. TRAF2, a tumor necrosis factor receptor-associated factor, interacts with complex II, modulating downstream apoptotic signaling (43). Studies have shown that the *FADD* gene is associated with critical genes such as LRRK2 and TRADD (21) (Fig. 8).

The protein encoded by the *FADD* gene functions as an adapter molecule that interacts with various cell surface receptors, that mediates apoptotic signaling. Through its C-terminal death domain, this protein is recruited by receptors such as TNFRSF6/FAS, TNFRSF25, and TNFSF10/TRAIL, thereby participating in the death signals initiated by these receptors. This interaction allows the N-terminal death effector domain of FADD to recruit and activate caspase-8, thereby initiating the cysteine protease cascade. Studies in mice have also highlighted the importance of this protein in the growth of primary T cells (44).

## Limitations

Although our study provides novel insights into the role of miR-361-5p in modulating *HMOX1* and *FADD* expression and its potential neuroprotective effect in an MPP ^+^ -induced PD cellular model, several limitations should be considered.

First, the validation of *HMOX1* and *FADD* at the protein level was not performed. Such confirmation, along with functional assays involving overexpression or knockdown of these proteins, would further strengthen the mechanistic link between miR-361-5p and neuronal cell death pathways. These experiments are planned as part of our future investigations.

Second, we employed undifferentiated SH-SY5Y neuroblastoma cells instead of primary dopaminergic neurons or differentiated cells, which may limit the physiological relevance of our findings. Third, additional assays exploring ferroptosis and mitochondrial morphology were not conducted, which could further validate the proposed mechanisms.

Future studies will also consider pharmacological modulation of the NRF2/HMOX1 axis (45) to expand the translational scope of this research.

## Conclusions

Overall, our findings demonstrate that downregulation of miR-361-5p leads to increased expression of HMOX1 and FADD in MPP^+^-treated SH-SY5Y cells, thereby enhancing oxidative stress and apoptosis. These results highlight the importance of miR-361-5p as a regulator of key pathogenic pathways in PD. HMOX1 and FADD may thus represent promising molecular targets for future therapeutic strategies.

## Experimental procedures

### Candidate miRNA selection

Literature mining was initially conducted to identify mitochondria-enriched miRNAs that are also deregulated in neurodegenerative and age-related disorders. For a molecular enrichment analysis of the miR-361-5p targetome and to identify the most significant pathways involved, validated target genes of miR-361-5p were obtained using the online database miRTarBase (Release 7.0) (http://mirtarbase.mbc.nctu.edu.tw/). Subsequently, DIANA miRPath (Release 2.0) (https://dianalab.e-ce.uth.gr/html/mirpathv3/index.php?r=mirpath) was employed to visualize the pathways related to the validated targets of miR-361-5p as a heatmap.

### miRNA target analysis

MiRNA target analysis involved the utilization of two databases, TargetScan (release 7.2) (http://www.targetscan.org/vert_72/) and miRWalk (release 2.0) (http://mirwalk.umm.uni-heidelberg.de/), to identify potential miR-361-5p targets in genomic sequences. In addition, deregulated genes in PD patients, compared to healthy controls, were identified using a human PD microarray dataset (GSE20186) available on NCBI Gene Expression Omnibus (GEO). The common predicted target genes from the human PD microarray dataset, TargetScan, and miRWalk were retrieved using the VENNY tool (Release 2.0) (https://bioinfogp.cnb.csic.es/tools/venny/). To ensure high-confidence targets and reduce the incidence of false positives, genes exhibiting an opposite expression pattern compared to miR-361-5p, along with high scores in prediction databases, were selected. Finally, a web-based tool for enrichment analysis (https://maayanlab.cloud/Enrichr/), was employed to confirm the involvement of potential target genes in significant signaling pathways correlated with PD. Experimental validation of selected target genes was further explored using miRTarBase (release 7.0) (http://mirtarbase.mbc.nctu.edu.tw/) and TarBase (release 8.0) (https://carolina.imis.athena-innovation.gr/diana_tools/web/index.php?r=tarbasev8%2Findex) databases.

### Cell culture

SH-SY5Y human neuroblastoma cells (Pasteur Institute) were cultured in Dulbecco's Modified Eagle Medium–Ham's Nutrient Mixture F-12 (DMEM/F12) medium (Gibco), supplemented with 10% fetal bovine serum (FBS) (Gibco) and 100 U/ml penicillin/streptomycin (Gibco). The cultured cells were maintained in a humidified incubator with 5% CO2 at 37 °C, and the media were refreshed every 3 days. Human embryonic kidney 293 (HEK293) cells were cultured in DMEM medium (Gibco) supplemented with 15% FBS (Gibco).

### Cell transfection

SH-SY5Y cells were cultured in 6-well dishes at a density of 2 × 10^4^ cells/cm^2^, reaching 80%-90% confluency before transfection. Transfection was carried out using 25 nM of miR-361-5p mimic and NC oligonucleotides (Dharmacon Inc), facilitated by lipofectamine 2000 reagent (Invitrogen) for 24 h.

### MPP^+^ treatment

After transfection, the cells were subjected to MPP^+^ treatment by replacing the basic medium with a treatment medium comprising DMEM, 1% FBS, and 2 mM MPP+ (Sigma-Aldrich) for 24 h to establish MPP + models.

### Cell viability assessment

After MPP^+^ treatment, cell viability was assessed using the MTS assay. SH-SY5Y cells were initially plated in 96-well plates until reaching 80% confluence. Subsequently, the cells were transfected with miR-361-5p mimics or NC oligonucleotides for 24 h. Following transfection, the cells were treated with 2 mM MPP^+^ for an additional 24 h.

For the MTS assay, 20 μl of MTS solution (Promega) was added to each well, and the cells were incubated for 4 h at 37 °C. The amount of produced formazan was then measured at a wavelength of 490 nm using an ELISA microplate reader (Awareness). All experiments were conducted in triplicate.

### Cell apoptosis assay

Cell apoptosis was assessed using the Annexin-V and propidium iodide staining kit (Mab Tag). After 48 h of transfection, both MPP^+^-treated and untreated (NC) cells were harvested, washed, and stained with 5 μl of Annexin-V conjugate and 5 μl of propidium iodide in the dark for 20 min. Apoptotic cells were quantified using a BD FACSCanto II Cell Analyzer (BD Biosciences) and analyzed with CellQuest Pro software (BD Biosciences).

### ROS assay

Intracellular ROS formation was quantified using the DCFH-DA assay. Following oligonucleotide transfection and MPP^+^ (2 mM) treatment, SH-SY5Y cells were washed with PBS and incubated with 0.5 μmol/L DCFH-DA (Sigma-Aldrich) at 37 °C for 20 min in the dark. The BD FACSCanto II Cell Analyzer (BD Biosciences) was then employed at a wavelength of 485 nm to measure cellular fluorescence, representing intracellular ROS levels.

### RNA extraction, cDNA synthesis, and RT-qPCR

To assess gene expression levels, total RNA was extracted from both MPP^+^-treated and untreated cells using TRIzol reagent (Invitrogen). The quality and quantity of the total RNA were evaluated using a NanoDrop 2000/2000c spectrophotometer (Thermo Fisher Scientific), measuring the 260/280 nm absorbance ratio.

For gene expression analysis, 1 μg of total RNA from each sample was reverse transcribed into complementary DNA (cDNA) using the cDNA Synthesis Kit (TaKaRa). The quantitative analysis of gene expression was then performed using SYBR Green Master Mix (TaKaRa) and gene-specific primers (Tables 1 and 2).

**Table 1:** RT-qPCR primers for mRNAs

mRNA	Primer name	Primer sequence
*HMOX1*	Forward	5′-CCCCAACGAAAAGCACATCCAG-3′
	Reverse	5′-GCTGCCACATTAGGGTGTCT-3′
*FADD*	Forward	5′-CTGTGTGCAGCATTTAACGTC-3′
	Reverse	5′-TATCTGTCCTCGATGCTGT-3′
*GAPDH*	Forward	3′-5′-CCACTCCTCCACCTTTGACG
	Reverse	5′-CCACCACCCTGTTGCTGTAG-3′
*U6 snRNA*	Forward	5′-CTCGCTTCGGCAGCACAT-3′
	Reverse	5′-TTTGCGTGTCATCCTTGCG-3′

**Table 2:** PCR primers for mRNAs

mRNA	Primer name	Primer sequence
*HMOX1*	Forward	5′-CTCGAGATGCAGGCATGCTGGCTC-3′
	Reverse	5′-GCGGCCGCTTCAAGCTACTATCAGAC-3′
*HMOX1-mut*	Forward	5′-GCGGCCGCCAAGCTACCAATGTTGTT-3′
	Reverse	5′-AAGCTTACGAGGTTGTTTTGGTGGTA-3′
*FADD*	Forward	CTCGAGCTACACAGCCTGGACTTTGGTT-3′-5′
	Reverse	5′-GCGGCCGCTAGTATGGAAGTTGTAAAGCTG-3′
*FADD-mut*	Forward	GCGGCCGCAAATCACTATCTTCAGAATTGCCAAGt-3′-5′
	Reverse	5′-AAGCTTACGAGGTTGTTTTGGTGGTA-3′
miR-361-5P	Forward	5′-AAGCTTACGAGGTTGTTTTGGTGGTA-3′
	Reverse	5′-GGATCCGTCTTAGTACTTCAGCGTGA-3′

For miR-361-5p expression analysis, 100 ng of total RNA was reverse transcribed using the miRCURY LNA Universal RT microRNA PCR kit (Exiqon). The relative expression of mRNAs and miRNAs was evaluated by the 2^−ΔΔCt^ method on an ABI PRISM 7500 instrument (Applied Biosystems) and normalized to the expression of glyceraldehyde 3-phosphate dehydrogenase (GAPDH) and U6 small nuclear RNA, respectively, as internal references.

### Dual-luciferase reporter assay

The dual-luciferase reporter assay was employed to elucidate the direct interaction between miR-361-5p and the 3′UTRs of predicted target genes.

Concurrently, the potential binding sites of miR-361-5p on its candidate target genes were predicted using the TargetScan database (http://www.targetscan.org). For the mutation assay, residues within the predicted seed regions were replaced with a thymidine heptamer to generate mutant sequences. Subsequently, synthesized fragments of both wild-type (WT) and mutant (MUT) target sites were inserted into the digested psiCHECK2 vectors (Promega). (All luciferase reporter constructs were confirmed by Sanger sequencing prior to use).

These luciferase reporter plasmids were co-transfected with miR-361-5p mimic or NC mimic into HEK293T cells and cultured for 48 h. Luciferase activity was assessed using a Dual-Luciferase Reporter Assay Kit on a Glomax Luminometer fluorescence detector (Promega). Each experiment was replicated in triplicate, and changes in Renilla luciferase activity were calculated relative to firefly luciferase enzyme activity (internal control).

### Statistical analysis

All experiments were performed with three independent biological replicates, each including technical replicates as indicated. All statistical analyses were conducted using GraphPad Prism 8 software. Data are presented as mean ± standard deviation (SD), and *p* values < 0.05 were considered statistically significant. Statistical comparisons among multiple groups were performed using one-way analysis of variance (t-tests and ANOVA).

## Data availability

All data generated or analyzed during this study are included in this published article and its supplementary information files.

## Conflict of interest

The authors declare that they have no conflicts of interest with the contents of this article.
